# Occupational health in the era of climate change and the green transition: a call for research

**DOI:** 10.1016/j.lanepe.2025.101353

**Published:** 2025-06-21

**Authors:** Michelle C. Turner, Xavier Basagaña, Maria Albin, Karin Broberg, Alex Burdorf, Kim R. van Daalen, Irina Guseva Canu, Henrik A. Kolstad, Manolis Kogevinas, Rachel Lowe, Neil Pearce, Frank Pega, Catherine Saget, Mary K. Schubauer-Berigan, Sara Svensson, Paolo Vineis, Kurt Straif

**Affiliations:** a Barcelona Institute for Global Health (ISGlobal), Barcelona, Spain; b Universitat Pompeu Fabra (UPF), Barcelona, Spain; c CIBER Epidemiología y Salud Pública (CIBERESP), Madrid, Spain; d Karolinska Institutet, Stockholm, Sweden; e Lund University, Lund, Sweden; f Erasmus MC, Rotterdam, the Netherlands; g Barcelona Supercomputing Center (BSC), Barcelona, Spain; h British Heart Foundation Cardiovascular Epidemiology Unit, Department of Public Health and Primary Care and Heart and Lung Research Institute, University of Cambridge, Cambridge, UK; i Department of Occupational and Environmental Health, Unisanté, University of Lausanne Faculty of Biology and Medicine, 1066, Lausanne, Vaud, Switzerland; j Aarhus University, Aarhus, Denmark; k Centre on Climate Change and Planetary Health and Centre for Mathematical Modelling of Infectious Diseases, London School of Hygiene and Tropical Medicine, London, UK; l Catalan Institution for Research and Advanced Studies (ICREA), Barcelona, Spain; m London School of Hygiene and Tropical Medicine (LSHTM), London, UK; n Department of Environment, Climate Change and Health, World Health Organization (WHO), Geneva, Switzerland; o International Labour Organization (ILO), Geneva, Switzerland; p International Agency for Research on Cancer (IARC), Lyon, France; q Halmstad University, Sweden; r MRC Centre for Environment and Health and NIHR GHRC on NCDs and Environmental Change, Imperial College, London, UK

**Keywords:** Circular economy, Climate change, Environment, Green transition, Occupation

## Abstract

Work and working conditions are fundamental social determinants of health. Climate change poses an urgent and growing threat to workers' health, through both direct exposure to environmental hazards and indirect exacerbation of social and health inequalities. Occupational health, which focusses on the promotion of mental and physical health and well-being of workers, is a key but often overlooked area in this context. Research at the intersection of climate change and occupational health remains limited. At the same time, climate change mitigation and adaptation efforts are driving rapid transformations in the workplace, including shifts towards sustainability and circular economy models. These transitions are creating new occupational hazards, including in renewable energy and circular economy sectors. We argue for increased investment in occupational health research and surveillance to address the evolving impacts of both climate change and the green transition, to better promote and protect workers’ health and rights.

## Introduction

Work and working conditions are fundamental underlying social determinants of health, and climate change is a rapidly emerging challenge impacting mental and physical health of workers on an unprecedented scale.^1^ Globally, 2024 was the warmest year on record, approximately 1.55 °C above pre-industrial levels.^2^^,^^3^ However, research on climate change and occupational health is currently limited, focusses largely on heat exposure, and represents only a small fraction of the available literature.^4^

As a consequence of climate change mitigation and adaptation efforts, massive transformations are occurring at the workplace.^5^ Economies are rapidly transforming towards sustainability, implementing new work practices, circular economy solutions, and expanding work in green jobs, with resulting novel workplace exposures and circumstances. Emerging occupational risks can clearly be identified for parts of the transition to renewable energy and for sectors in the circular economy. However, there is relatively little research on these effects. The field of occupational health focusses on the promotion of mental and physical health and well-being of workers. Workers have human rights to health; a clean, healthy, and sustainable environment; and a safe and healthy working environment; however, workers are often among the first and greatest exposed to hazards.^1^^,^^6^^,^^7^

Following a brief overview, we describe specific emerging epidemiological knowledge gaps and research needs in selected prominent, yet under-researched, occupational sectors of interest relevant to climate change (emergency work in firefighting), the green economy (renewable energy and wind turbine manufacturing, waste management and recycling) and the transition towards more sustainable work practices (health care, transport and mobility). We seek to demonstrate that there has been insufficient attention to this area. There are also implications for official worker monitoring systems which must respond to capture new indicators of health and climate data.^8^^,^^9^

## Overview of climate change and the green transition for worker health

Climate change may affect workers through increased exposure to a range of hazards including heat, extreme weather, outdoor air pollution, drought, wildfires, allergens, vector-borne diseases, and their combined exposures.^2^^,^^6^^,^^10^, ^11^, ^12^ There are approximately 2.41 billion workers exposed to excessive heat, representing 70% of the working population.^6^ Trends in excessive heat exposure over the past two decades in Europe and Central Asia increased by 17.3%, nearly twice the global average.^2^^,^^12^ Worldwide a total of 18,970 work-related heat deaths occur each year as well as 22.85 million injuries.^6^^,^^12^ There are also adverse impacts of occupational heat on performance and productivity,^8^ mental health and psychosocial outcomes.^12^ There is a substantial risk severity for heat stress among outdoor workers in Southern Europe, increasing to critical severity by the mid-to late-century.^2^

Workers most impacted by climate change typically include those in outdoor occupations such as in construction, agriculture, fishing, forestry, firefighting and emergency services, transportation and tourism.^6^^,^^10^^,^^12^^,^^13^ Workers are predominantly younger males, oftentimes self-employed with seasonal work and of lower socioeconomic status.^13^ Indoor workers with local heat sources or in non-climate-controlled conditions are also affected.^10^ Low and middle-income countries (LMICs) have contributed the least greenhouse gas (GHG) emissions and are frequently the most adversely affected.^11^

Indirect impacts of climate change, including job disruption and displacement, are far-reaching, impacting life trajectories of workers and their families. Increases in unemployment due to climate change are predicted, in particular among males, and in middle-income countries,^14^ as are increases in climate-induced migration.^15^ There may also be maladaptation with unintended consequences.^11^^,^^13^

Climate change mitigation policies must be rapid.^16^ They include phasing-out fossil fuels while simultaneously phasing-in renewable energy. The European Green Deal aims to transform the economy, decoupling growth from resource use to be the first climate-neutral continent as a binding commitment.^17^ It plans a number of ambitious activities covering all relevant sectors of the economy, including exploiting low-carbon and low-pollutant solutions, implementing new technologies in energy production, distribution and end use, clean transport, and adapting industrial activities and consumption patterns to a circular economy, among others.

In 2022, energy production (electricity, gas, steam, air conditioning) accounted for 26% of GHG emissions in Europe and 1% of employment.^13^ Decarbonisation of electricity generation has had the greatest impact in terms of carbon mitigation intensity among interventions in any sector.^16^ Renewable energy sources however, represent only 24.5% of total energy consumption.^18^ Mean levels of GHG emissions per worker in the EU have declined since 2010 and are currently at their lowest value.^13^ However, the pace of decarbonisation remains inadequate, with Europe estimated to reach climate neutrality by 2100, far beyond the 2050 target.^17^^,^^19^

Forty percent of workers in the EU are expected to be impacted by the green transition.^13^ There are new occupations that are emerging and existing occupations that require enhanced skills; other occupations will be eliminated. Many activities that contribute to GHG emissions also entail serious occupational risks (i.e., coal mining/combustion). Most recently, automotive gasoline was classified as an International Agency for Research on Cancer (IARC) Group 1 human carcinogen, causing bladder cancer and acute myeloid leukemia, with consistent increases in cancer incidence observed in occupational studies of service station attendants and gasoline distribution workers and in studies assessing gasoline exposure.^20^ Replacement of hazardous jobs should clearly be supported. Globally, the green energy transition is predicted to create 24 million jobs by 2030 and abolish four million related to fossil fuels and in parallel the circular economy to create 77 million jobs while making 71 million redundant.^21^

**Fig. 1:**
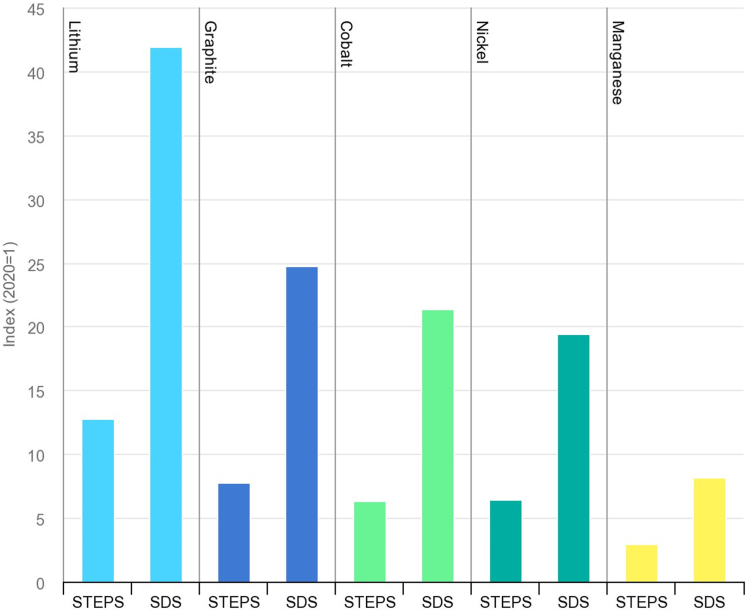
Growth in demand for selected battery-related minerals from clean energy technologies in 2040 relative to 2020 levels by scenario (STEPS, Stated Policies Scenario; SDS, Sustainable Development Scenario) (Licence: CC BY 4.0).^22^

While the green transition will reduce or eliminate many dangerous exposures as a co-benefit, it will also introduce new hazards, as well as spread of toxic critical raw materials (e.g., lithium, cobalt, nickel; Fig. 1) in new sectors.^22^ The transition will require development of new materials for catalysts, batteries, replacement of plastics, and other emerging applications. The potential risks are understudied. There may be recirculation of building materials with known toxicants (e.g., respirable crystalline silica, asbestos, polychlorinated biphenyls), especially in small and medium-sized businesses.^23^

**Fig. 2:**
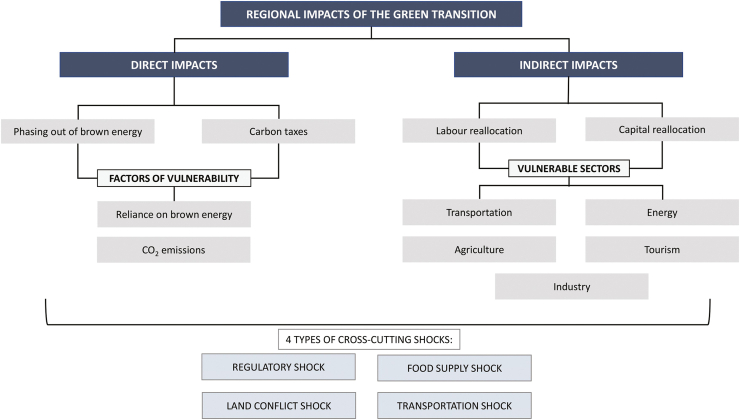
Schematic representation of the theoretical framework for the identification and assessment of the regional impacts of the green transition (Licence: CC BY 4.0).^25^

Foresight scenarios highlight several challenges.^24^ The best scenario considers reaching a fully circular economy in which dangerous and polluting jobs have disappeared, and upskilling and reskilling has permitted workers to fill new safe jobs. The worst scenario describes a transition which has stagnated amid economic and environmental crises and heightened social tensions. Intermediate alternatives include achieving a carbon neutral/circular economy but at a price of worker safety or increasing regional divides. There may be green discontent and regional differences in opportunities and threats regarding changes in the geography of jobs and wealth (Fig. 2).^25^ Employment transitions may exacerbate persistent gender-based inequities.^5^^,^^6^

Occupational health researchers can support the green transition as a necessary change, while simultaneously promoting investigation of associated hazards. Areas such as energy transition, waste management and the circular economy, have typically been under-researched in terms of health.^26^ For new green jobs, it is often presumed that they will be of higher quality, though little evidence exists. Occupational research is relevant for general populations. For carcinogens, occupational studies led to identification of hazards also present, at generally lower levels, in the general environment and to a search for solutions.^27^ The same may occur for research on climate change and the green economy.

## Knowledge gaps and research needs among specific sectors

### Emergency work in firefighting

Emergency service workers are affected by increases in the frequency and intensity of extreme weather-related events, including wildfires in temperate areas with urban settlements expanding into wildland.^28^ Increases in wildfires impact firefighters. There are more than 15 million firefighters globally,^29^ though data gaps exist on the number of firefighters responding to wildland fires. Among municipal fire departments in the US, 86% are involved in wildfire responses.^30^ The number of volunteer firefighters often exceeds paid firefighters.

Firefighters experience many potentially hazardous exposures including combustion products from fires [e.g., polycyclic aromatic hydrocarbons, aromatic amines, aldehydes, particulate matter (PM), persistent organic pollutants, heavy metals], building materials (e.g., asbestos, silica, flame retardants), perfluorinated compounds, excessive noise and heat.^31^ Working conditions include long working hours, night shift work, physical and emotional demands.

Wildfires involve combustion of organic material.^32^ Due to encroachment of settlements into wildland areas, firefighters working at the wildland–urban interface may have exposures common to both settings. Concentrations of PM <2.5 μm in diameter have averaged between 0.25 and 1.0 mg/m^3^ among wildland firefighters.^31^ Firefighters working in wildlands or at the wildland–urban interface are often volunteers with few resources to protect against harmful exposures.^31^ They may have lengthy deployments and restricted access to effective decontamination.^31^ They typically do not wear respiratory protection, and clothing often provides minimal protection.^33^ Outdoor workers are also exposed to wildfire smoke. Some regulations exist to protect outdoor workers from wildfire smoke exposure.^33^

Occupational exposure as a firefighter was recently classified as “carcinogenic to humans” (IARC Group 1).^31^ There was “sufficient” evidence of carcinogenicity for mesothelioma and bladder cancer and “limited” evidence for skin melanoma, non-Hodgkin lymphoma, and cancers of the colon, prostate, and testis. There was “strong” mechanistic evidence based on five “key characteristics of carcinogens” (KCCs) among firefighters: genotoxicity, epigenetic alterations, oxidative stress, chronic inflammation, and modulation of receptor-mediated effects. These effects may also be relevant to other chronic diseases, e.g., respiratory, cardiovascular.^33^ Although few studies evaluated cancer risk in populations with a large proportion of wildland firefighters, many of the studies of KCCs were conducted among wildland firefighters, with similar findings to municipal firefighters.

Major information gaps that were noted included studies of cancer risk among wildland firefighters and firefighters at the wildland–urban interface, systematic documentation of the numbers of wildland fires and firefighters, particularly in LMICs, real-time exposure measurements and chemical composition analysis, studies considering sex, hydration, exposure route, tasks, or biological half-life when evaluating biomarkers of exposure, and the use and effectiveness of protective clothing.^31^ Impacts of physical stressors on carcinogenesis are unclear, though heat strain may impact thermoregulatory processes related to immune response.^31^ The U.S. National Institute for Occupational Safety and Health proposed a nationwide registry of firefighters, including in wildland settings, with emphasis on inclusion of women and ethnic minorities.^34^ The future development of the registry is currently uncertain.^35^ A protocol for a U.S. Fire Fighter Cancer Cohort Study (FFCCS) was recently published.^36^ Vulnerable and undocumented workers may be involved in clean-up following climate disasters.

### Renewable energy and wind turbine manufacturing

Globally, in 2023, there were at least 16.2 million workers in renewable energy jobs, an increase from 13.7 million in 2022.^5^ The majority of jobs (7.4 million) were in China, while in Europe there were 1.8 million. Little information is available on the diversity of the workforce; however, women are under-represented. The EU aims for 50% of its electricity from wind by 2050.^37^ The revised European Renewable Energy Directive sets a binding target of 42.5% of energy use from renewable energy sources by 2030, aiming at 45%.^38^ This would require capacity to increase to more than 500 GW from 204 GW in 2022. The wind energy sector employed 316,300 workers in Europe in 2023 with 100,000 additional workers needed by 2030.^5^^,^^38^

Workers in the wind energy sector are exposed to particular occupational risks. Production, installation, and maintenance of wind turbines encompass manually demanding tasks with increased risks of musculoskeletal disorders of the upper extremities. During the production of wind turbine blades, workers can be exposed to high levels of styrene evaporating from fiberglass reinforced polyester composites and, during finishing, to epoxy resins through dermal contact. Styrene is neurotoxic^39^ and has been classified in IARC Group 2A (“probably carcinogenic to humans”).^40^ Epoxy components are well documented skin sensitizers and causes of dermatitis, occupational asthma and hypersensitivity pneumonitis. Workers in the fiberglass reinforced polyester industry are at increased occurrence of obstructive lung function impairment and pulmonary inflammation.

Styrene exposure can be mitigated, but not eliminated, by ventilation, respirators, and new production processes. Wind turbine workers remain at manifold increased risk of skin sensitization and dermatitis despite comprehensive skin protection.^41^ Most decommissioned wind turbine blades are currently landfilled. Although an increasing proportion are recycled, little is known about potential adverse exposure characteristics and health impacts of such recycling work.^42^ Further research is needed to clarify whether styrene is carcinogenic to humans, e.g., by pooling existing cohorts from the wind turbine and other styrene exposed industries. Developing monitoring programs for skin contamination and sensitization among epoxy exposed wind turbine workers is needed as is collecting long-term follow-up data regarding psychosocial risks and emotional demands in their operation and maintenance.^43^

There is some knowledge about respiratory health effects of microbial exposures in the green energy sector.^44^ The green energy transition also results in increasing demand of metals. There is comprehensive documentation of extreme working conditions and violation of human rights during artisanal and small scale mining of cobalt (metallic cobalt has been classified in IARC Group 2A)^45^ and other toxic metals for lithium batteries.^46^ Solar voltaic panels may contain metals such as cadmium and lead,^47^ but the principal component of the panels is silicon extracted from high purity quartz. Crystalline silica (in the form of quartz) is a recognized IARC Group 1 lung carcinogen, also at low exposure levels, and is a well-documented cause of silicosis. The production of photovoltaic panels from silicon shares characteristics with the production of other semi-conductors, involves a wide range of toxic materials, but the production is to a large extent automatized or occurs in clean rooms with low exposure levels. There are safety hazards among solar energy workers including solar ultraviolet (UV) radiation exposure, falls from heights (i.e., rooftop installers with limited walking space), musculoskeletal disorders, and burn hazards.^48^ Little is known about health hazards in the geothermal, hydropower, or energy storage industry from mining to decommissioning and recycling.^6^^,^^49^

### Waste management and recycling

Investments in recycling aim to reduce the amount of hazardous substances in the environment as well as reuse scarce critical raw materials. The European Circular Economy Action Plan aims to maintain resources in the economy to the greatest extent possible.^50^ Over 4.2 million workers in Europe were employed in the circular economy in 2021 (recycling, repair, reuse), an increase from 3.3 million in 2005.^51^ However, increased recycling may lead to hazardous exposure to workers. Recycling work is complex, includes formal and informal work, ranges from collection of domestic waste to recycling pure metals, and uses manual to highly automated systems. In parallel to increases in formal recycling work, there has also been a proliferation of informal recycling and waste work.^52^

Recycling carries risks including of high exposure to bioaerosols and infectious agents, mixed toxic metals and organic chemicals, aerosols from fires, repetitive work, noise, and injuries.^13^^,^^53^ Several surveys have examined the working environment in the recycling industry.^53^ Elevated levels of toxic metals, perfluorinated compounds and phthalates have been found in blood and urine from workers who recycle metal goods, batteries or plastics.^53^ Recycling of domestic waste generates bioaerosols that can cause respiratory and systemic health effects. Impaired lung function, and cardiac and pulmonary, gastrointestinal, eye, skin and musculoskeletal symptoms have been found in workers sorting and recycling e-waste and plastics.^53^ Injuries have been reported among informal waste pickers.^54^

Little is known about bioaerosol exposure-response relations and the role of specific microbial species in inflammation. A recent study reported increased post-shift serum concentrations of inflammatory markers among recycling workers.^55^ Waste is highly diverse and continuously changing due to the high flow of new chemicals and products. Comprehensive, non-targeted exposure characterisation, including mixtures, is lacking, which limits exposure assessment. Awkward body postures and repetitive work during recycling have been described.^56^ However, there is a lack of high-quality studies and investigation in the sector. There are few scientific investigations on prevalence, determinants, and prevention of injuries. Studies focusing on organization of work, personal protection, and proactive health and safety management are sparse and mainly limited to recycling of e-waste.^57^ E-waste work was recently accorded “high” priority for evaluation by the IARC Monographs on the basis of mechanistic evidence in exposed humans; though there are few studies of long-term exposures and no evidence regarding cancer risk in epidemiological studies.^58^

### Health care

Climate change confronts the health care sector with a dual challenge. Climate change leads to increased pressure on health system capacity.^2^^,^^6^ Simultaneously, the health care sector is globally responsible for about 4.6% of GHG emissions.^59^ Pharmaceuticals are among the largest contributor to the carbon footprint of the sector, followed by use of disposable products with multiple layers of packaging, waste production, and material-intensive medical devices.^60^ The health community is central to climate leadership and decarbonisation of healthcare systems.^61^

In many health care organizations, interventions are developed to facilitate transition towards more sustainable health care with less GHG emissions by changing work processes and content. However, it has been hypothesized that environmental sustainability may be in tension with quality of work and satisfaction of healthcare providers (e.g. increased work pressure), but could also be a potential lever for these (e.g. engagement).^62^ Sustainability can be included as a separate (fifth) domain of quality in health care.^63^ This builds upon the well-established quadruple aim theory, which proposes that health and wellbeing of personnel is a fourth essential value, next to three classical values of effectiveness of care, reasonable costs, and sufficient improvements in quality of life of patients. The theory was propelled by empirical evidence that work pressure adversely affects mental health of health care personnel resulting in suboptimal quality and medical errors. Medical errors increase mental health issues, such as burnout.^64^

With this theory in mind, it is important to evaluate how introduction of sustainable work practices will impact quality of work of health care personnel, and their health and wellbeing. Introduction of new sustainable work practices cannot be evaluated in randomized controlled trials, since transformation towards sustainable health care is a complex process with many interacting stakeholders, and as such must be evaluated by ‘natural experiments’ with observational data, taking advantage of large variation in uptake and magnitude of interventions in real settings. Research findings would offer new possibilities to promote individual and group-based resilience in health care to ensure that environmental sustainability does not create additional work pressure but contributes to quality of work and mental health of health care workers.

### Transport and mobility

The transportation sector is estimated to be responsible for approximately 25% of the total net anthropogenic GHG emissions in Europe and employs 10 million workers.^65^ A 90% reduction in transport related GHG emissions by 2050 is sought through various policies including increasing cleaner fuels and renewable energy sources.^17^ Since 1990, emissions of air pollutants have decreased for all transport modes, except for shipping and aviation.^66^ Public transportation, compared to private cars, is less polluting and more energy efficient.^16^ However, private car trips still form the majority (73.0%) of all passenger transport in the EU-27, whereas public transport 15.6%.^67^

Many European cities have implemented measures to encourage use of hybrid and electric buses. Public transport operators are also looking for additional solutions to drive buses at their maximum efficiency. Eco-driving is defined as “*an easy and cost-efficient way to reduce fuel consumption, GHGs and accident rates, but also an attitude and respect for society as a whole*”.^68^ However, understanding of eco-driving effectiveness is limited. While short-term studies show that eco-driving training can lead to reductions in fuel consumption, longer-term impacts are less clear. Eco-driving assistance systems (EDAS), installed in public buses, may also reduce fuel consumption, however they may not be acceptable to drivers due to the psychological stress of being monitored.^69^ EDAS comprise of additional sensors on the bus that detect driving behaviour and contextual information with driving quality scores reported to the drivers. Stress may increase when the driver is unable to drive in an eco-friendly manner due to traffic intensity and flow, weather conditions, or heating or air conditioning use. The definition of an innovative transport system, which should combine “*passengers' desire for mobility, according to their own specific needs and expectations, the operators' economic requirements, and at the same time meeting general environmental needs*”^70^ neglects driver's needs.^71^

Transport workers are exposed to a range of physical, chemical, psychosocial and biological hazards.^13^ Interruptions in public transport services due to climate change also impact population health. Higher temperatures result in more difficult driving conditions, higher mental load and cognitive demands, and reduced reaction times. Interventions are needed to mitigate physical and psychosocial risks, the demographic transition, and increasing shortage of drivers.

## Global monitoring initiatives

Several monitoring initiatives have sought to quantify various aspects of current and future climate change occupational impacts to inform research and policy-making priorities.^9^ It is important that global monitoring initiatives are based on the highest quality data. However, in many cases, monitoring data are insufficient or inaccessible. Challenges in strengthening worker monitoring systems have been described.^9^ There are additional complexities when estimating likely impacts of climate change on burdens of occupational and work-related diseases. We need not only estimates for current exposures, but also how they are likely to change over time and impacts on disease burden. The health impacts of climate change are unequally distributed, disproportionately burdening already disadvantaged workers. Growing health inequities and social injustices are expected. Predicting these changes requires considerably more data than are available.

### WHO burden of disease estimates

Since the 1990s, the WHO Comparative Risk Assessment quantifies global, regional, and national burdens of disease by risk factor, cause, sex and age group. WHO's global burden of disease estimates attributed 0.2 million deaths and six million disability-adjusted life years (DALYs) to selected climate change risk factors in 2000^72^ and 1.9 million deaths and 90 million DALYs to selected occupational risk factors in 2016.^73^ Estimates are lacking of the burden of disease attributable to occupational climate change exposures. WHO has provided estimates of the population exposed to climate change while working outdoors, estimating that there were 1.6 billion outdoor workers globally in 2023, or 25.9% of the working population.^8^ WHO and ILO attributed 18,960 deaths (uncertainty range (UR) 18,180–19,740) and 0.5 million DALYs (UR 0.4–0.5) from non-melanoma skin cancer to occupational exposure to solar UV radiation.^74^

Official estimates and indicators of the burden of disease from occupational exposures to climate change risk factors is a global priority for workers’ health monitoring.^9^ To produce these, primary and synthesised data and evidence are needed on: occupational exposures to climate change risk factors; mechanisms linking them to relevant health outcomes for hazard identification; and their effect on these health outcomes for risk quantification. Although there is extensive literature on hot and cold temperatures and occupational injury,^75^ less is known regarding occupational disease. These data should also be disaggregated by sex, age, and other socio-economic variables to enable health inequality analysis. Only 19% of the total 1.9 million work-related deaths in 2016 were due to injuries, while 81% were due to disease.^76^ The toolbox for estimating occupational disease burden must be expanded to include nowcasting and forecasting methods, and better methods to address uncertainties in input parameters, learning from climate science. WHO is developing the Research for Action on Climate Change and Health agenda—REACH 2035, which may include a research agenda on occupational health.

### ILO global reports on occupational heat stress

ILO predicted that, in 2030, 2.2% of global labour productivity will be lost due to heat stress compared with 1.4% in 1995, particularly in countries with weaker occupational safety and health frameworks.^77^ Southern and East Asia and Western Africa would lose between 4 and 8% of working hours. New strengthened occupational heat regulations are needed.^12^ A substantial proportion (51.8%) of global climate change mitigation costs could be offset by economic benefits due to avoided labour productivity losses alone.^78^ Labour productivity benefits of air quality improvements due to climate change mitigation actions are realised more rapidly to those of reduced heat exposure.^79^

Further research is needed to analyse the impact of climate change on labour market inequality. For example, older workers may be more likely to work in some outdoor occupations, such as street vendors, and are vulnerable to risks of heat-related illness and related labour productivity losses.^80^^,^^81^ Younger workers may be more vulnerable to heat-related occupational injuries.^75^^,^^81^ Few studies have examined contextual modifiers of temperature-injury associations. Adaptation of workers over time with respect to heat-related injury risk was observed in Spain.^75^ A detailed mapping of adaptation measures to prevent labour productivity losses around the world is lacking.

Preventive strategies should be sustainable and based upon scientific evidence, though their effectiveness has rarely been investigated in terms of health outcomes.^6^^,^^10^^,^^12^^,^^77^ Occupational heat stress warning systems that provide tailored recommendations to workers are being advanced.^6^^,^^82^ Further studies are needed considering gendered adaptation strategies based on social and cultural norms.^11^

### The Lancet Countdown Europe

The Lancet Countdown Europe monitors and quantifies health impacts of climate change and health co-benefits of accelerated mitigation and adaptation using over forty indicators, adding nine new indicators from 2022.^19^ The 2024 indicators continued to highlight accelerating trends in health-related hazards, exposures, vulnerabilities and risks and insufficiently ambitious mitigation and adaptation strategies. For example, clinically relevant pollen seasons are starting earlier, climatic suitability for a range of infectious diseases is rapidly increasing, and heat-related mortality and morbidity are increasing. Many such health risks are paired with substantial occupational impacts and economic losses, such as reduced labour supply in occupations with high heat exposure (agriculture, forestry, mining, quarrying and construction), particularly in Southern Europe, and reduced gross domestic product per capita growth. There were also positive trends in wildfire danger. Twenty-nine of 53 countries provide net subsidies for fossil fuels. Analysis of the scientific literature showed a small amount on mitigation and adaptation topics, as well as of equality, equity or justice. There were few references to the intersection of climate change and health terms in legislators’ speeches in the European Parliament though there was high engagement in corporate annual reports.

Indicators often depend on publicly available data collected for other purposes. As a result, absence of (standardised) disaggregated health burden and population data often forms a barrier to uncovering climate-related health disparities and inequalities.^83^ Similarly, whilst many EU countries tend to have a wealth of data available—coverage of Eastern European countries is often weaker. Due to lack of accessible occupational health data, the indicator framework is currently limited in its ability to track climate change impacts on occupational health. New indicators relevant to diverse dimensions of occupational health are needed.

## Conclusions

There is an urgent need to rapidly move towards increased sustainability. The speed in collecting and synthesizing necessary data for worker protection needs to match the speed in transition to renewable energy and the circular economy. Occupational health research responded rapidly to other recent crises (e.g., COVID-19). Re-orienting existing research infrastructures can facilitate a more rapid research transition.^84^ Intervention-based studies across multiple sectors are needed to support healthy and liveable working environments.^1^^,^^85^^,^^86^ Contact with those developing new technologies may promote “safe and sustainable by design” focussing future research on those most likely implemented in practice. Beyond epidemiological research, solution-oriented studies drawing upon transdisciplinary expert networks in impacted sectors are needed.^26^ Finally, as recent global economic and political trends threaten the green transition and policy packages,^35^^,^^87^ researchers can promote greater investment in occupational research, academic freedoms, and transparency in higher education, including regarding obstruction of climate action.^88^

Search strategy and selection criteria: References for this Personal View were identified through searches of PubMed until April, 2025. In addition, searches in the grey literature were performed and articles were identified in the authors’ own files. We considered original studies, reviews, authoritative reports and databases in English language. We sought to highlight key contributions relevant to climate change, the green transition and occupational health. References included here were selected on the basis of being recent and informative to the discussion of research needs.

## Contributors

MCT, XB, KS conceived of the manuscript and acquired funding. All authors drafted text, revised the manuscript, and approved the final version.

## Declaration of interests

The authors have no conflict of interest to declare.

## Appendix A Supplementary data

Translated Abstracts
