# Supplementary material for: Occupational health in the era of climate change and the green transition: a call for research
Source: Lancet Reg Health Eur. 2025 Jun 21;54:101353. doi: 10.1016/j.lanepe.2025.101353 (PMC12226071; doi:10.1016/j.lanepe.2025.101353)
Supplement: Translated Abstracts [file mmc1.docx]

**Occupational health in the era of climate change and the green transition: a call for research**

**Translated abstracts**

**Catalan**

El treball i les condicions laborals són determinants socials fonamentals de la salut. El canvi climàtic és una amenaça creixent i urgent per la salut dels treballadors, de manera directa a través de l’exposició a riscos ambientals, i de manera indirecta ampliant les desigualtats socials i en salut. La salut ocupacional, que es focalitza en la promoció de la salut mental i física i del benestar dels treballadors, és una àrea clau en aquest context, tot i que freqüentment oblidada. La recerca en la intersecció del canvi climàtic i la salut ocupacional és encara limitada. En paral·lel, els llocs de treball estan patint grans transformacions com a conseqüència dels esforços de mitigació i adaptació al canvi climàtic, com ara els relacionats amb la millora de la sostenibilitat i la transició cap a l’economia circular. Cal incrementar la investigació i la vigilància per avaluar l’evolució dels impactes del canvi climàtic i la transició ecològica, per així millorar la promoció i la protecció de la salut i els drets dels treballadors.

**Danish**

Arbejde og arbejdsforhold er grundlæggende sociale determinanter for sundhed. Klimaændringer udgør en akut og voksende trussel mod arbejderes sundhed, både via direkte eksponering for miljømæssige farer og indirekte via forværring af social og sundhedsmæssig ulighed. Et arbejdsmiljø, som fokuserer på at fremme arbejderes mentale og fysiske sundhed og trivsel, er i denne sammenhæng et centralt, men ofte overset område. Forskning i krydsfeltet mellem klimaændringer og arbejdsmiljø er fortsat begrænset. Samtidig medfører indsatser overfor klimaændringer hurtige ændringer på arbejdspladserne, herunder skift mod bæredygtighed og cirkulær økonomi. Disse ændringer skaber nye erhvervsmæssige risici, blandt andet inden for vedvarende energi. Vi argumenterer for øgede investeringer i forskning og overvågning af arbejdsmiljøet for at kunne håndtere konsekvenserne af både klimaændringer og den grønne omstilling, så vi bedre kan fremme og beskytte arbejderes sundhed og rettigheder.

**Dutch**

Werk en arbeidsomstandigheden zijn fundamentele sociale determinanten van gezondheid. Klimaatverandering vormt een urgente en toenemende bedreiging voor de gezondheid van werknemers, zowel door directe blootstelling aan milieugevaren als door indirecte toename van sociale en gezondheidsongelijkheden. Arbeidsgezondheid, die gericht is op het bevorderen van de mentale en fysieke gezondheid en het welzijn van werknemers, is een essentieel maar vaak over het hoofd gezien aspect in deze context. Onderzoek op het snijvlak van klimaatverandering en arbeidsgezondheid is tot op heden beperkt. Tegelijkertijd leiden mitigatie- en adaptatiemaatregelen met betrekking tot klimaatverandering tot ingrijpende transformaties op de werkvloer, waaronder een transitie naar duurzame en circulaire economische modellen. Deze transities brengen nieuwe arbeid gerelateerde risico’s met zich mee, ook in sectoren zoals hernieuwbare energie en de circulaire economie. Wij benadrukken de noodzaak voor meer investering in arbeidsgezondheid onderzoek en monitoring om de veranderende impact van zowel klimaatverandering als de groene transitie aan te pakken, en ter bevordering van de bescherming van werknemersrechten en -welzijn.

**French**

Le travail et les conditions de travail sont des déterminants sociaux fondamentaux de la santé. Le changement climatique représente une menace urgente et croissante pour la santé des travailleurs, tant par l'exposition directe aux risques environnementaux que par l'aggravation indirecte des inégalités sociales et de santé. La santé au travail, qui se concentre sur la promotion de la santé mentale et physique et du bien-être des travailleurs, est un domaine clé mais souvent négligé dans ce contexte. Les recherches à l'intersection du changement climatique et de la santé au travail restent limitées. Dans le même temps, les efforts d’atténuation et d’adaptation au changement climatique entraînent des transformations rapides sur le lieu de travail, notamment des évolutions vers des modèles de durabilité et d’économie circulaire. Ces transitions créent de nouveaux dangers professionnels, notamment dans les secteurs des énergies renouvelables et de l'économie circulaire. Nous plaidons en faveur d'un investissement accru dans la recherche et la surveillance épidémiologique en santé au travail pour faire face aux impacts évolutifs du changement climatique et de la transition écologique, et pour mieux promouvoir et protéger la santé et les droits des travailleurs.

**German**

Arbeit und Arbeitsbedingungen sind grundlegende soziale Determinanten von Gesundheit. Der Klimawandel stellt eine dringende und wachsende Bedrohung für die Gesundheit von Arbeitnehmer*innen dar – sowohl durch direkte Umweltgefahren als auch durch die indirekte Verschärfung sozialer und gesundheitlicher Ungleichheiten. Die Arbeitsmedizin, die sich auf die Förderung der mentalen und physischen Gesundheit sowie des Wohlbefindens von Beschäftigten konzentriert, ist in diesem Zusammenhang ein zentrales, aber häufig zu wenig beachtetes Feld. Die Forschung an der Schnittstelle von Klimawandel und Arbeitsschutz ist nach wie vor begrenzt. Gleichzeitig führen Klimaschutz- und Anpassungsmaßnahmen durch den Umstieg auf nachhaltige und zirkuläre Wirtschaftsmodelle zu tiefgreifenden Veränderungen in der Arbeitswelt. Diese Übergänge bringen neue arbeitsbezogene Risiken mit sich – auch in Sektoren wie erneuerbare Energien und Kreislaufwirtschaft. Wir plädieren für verstärkte Investitionen in Forschung und Überwachung im Bereich der Arbeitsmedizin, um die sich wandelnden Auswirkungen des Klimawandels und der grünen Transformation besser zu erfassen und die Gesundheit und Rechte der Arbeitnehmer*innen gezielter zu fördern und zu schützen.

**Spanish**

El trabajo y las condiciones laborales son determinantes sociales fundamentales de la salud. El cambio climático es una amenaza creciente y urgente para la salud de los trabajadores, de manera directa a través de la exposición a riesgos ambientales y de manera indirecta ampliando las desigualdades sociales y en salud. La salud ocupacional, que se focaliza en la promoción de la salud mental y física y del bienestar de los trabajadores, es un área clave en este contexto, aunque frecuentemente olvidada. La investigación en la intersección del cambio climático y la salud ocupacional es todavía limitada. Paralelamente, los lugares de trabajo sufren grandes transformaciones como consecuencia de los esfuerzos de mitigación y adaptación al cambio climático, como la mejora de la sostenibilidad y la transición a la economía circular. Estas transformaciones resultan en nuevos riesgos laborales, por ejemplo en los sectores de las energías renovables y de la economía circular. Es necesario incrementar la investigación y la vigilancia para evaluar la evolución de los impactos del cambio climático y la transición ecológica, para mejorar así la promoción y la protección de la salud y los derechos de los trabajadores.

**Swedish**

Klimatförändringar utgör ett direkt och ökande hot mot hälsan i den arbetande befolkningen. Arbetshälsa, som fokuserar på främjande av både den mentala och fysiska hälsan hos arbetstagare, är en viktig men ofta negligerad del av detta. Forskningen om klimatförändringar och arbetshälsa är fortfarande i sin linda. Parallellt pågår en massiv omställning på arbetsplatserna för att motverka klimatförändringen och anpassa verksamheten till den. Ekonomin ställer om mot mer hållbara och cirkulära lösningar, vilket ger snabbt förändrade och nya exponeringar på arbetsplatserna. Framväxande arbetsmiljörisker kan klart identifieras för delar av omställningen till förnybar energi och för vissa sektorer i den cirkulära ekonomin. Vi belyser behovet av ökad satsning på forskning om arbetshälsa relaterad till klimatförändring och grön omställning för att förbättra arbetarskyddet och främja hälsosamma arbetsförhållanden.
